# The Valdivia Project: genetic characterization of a unique Wolfram Syndrome cluster in coastal Ecuador

**DOI:** 10.1186/s13023-026-04344-z

**Published:** 2026-04-17

**Authors:** Josefa E. Palacio, María S. Verdesoto, Noemí L. Bautista, Abigail J. Segura, Roberto C. Cedeño, Nancy J. Flores, Juan C. Zevallos

**Affiliations:** Universidad Espíritu Santo, Facultad de Ciencias de la Salud, Samborondón, Ecuador

**Keywords:** Wolfram syndrome, Type 1 diabetes, Optic atrophy, Sensorineural deafness, New variant

## Abstract

**Objective:**

This study aims to document the first genetically confirmed cases of WS1 in Ecuador.

**Design and methods:**

A Cross-Sectional study was conducted among individuals with early-onset insulin-dependent diabetes, residing in Santa Elena Province, Ecuador. A detailed clinical history and family tree were obtained along with laboratory and imaging diagnostics. A genetic whole exome sequencing was performed to confirm WS1 or not (NWS).

**Results:**

This Ecuadorian cohort included 38 participants (19 males) aged < 35 years with an average diabetes onset at 3 years old. Approximately 50% of the patients are related by consanguinity. Genetic studies tested positive for WS1 in 26 (69%) patients, which yields a prevalence of 1/12,000 inhabitants and the presence of two previously undescribed variants located in exon 8. Twenty-three were homozygous. Positive antibody testing was reported in 3/26 WS1 and in 4/11 NWS patients. High prevalence of severe and early neurological complications: optic atrophy, deafness and urinary disorders were documented. No patient had diabetes insipidus.

**Conclusions:**

Findings suggest the highest WS1 prevalence worldwide and two novel WS1 variants. There is high consanguinity and frequency of severe, early clinical complications among WS1 patients. In addition, only 11% were diagnosed with type 1 diabetes, suggesting that younger patients with early-onset, non-autoimmune diabetes should undergo comprehensive genetic testing.

## Highlights

Why did we undertake this study?

What is the specific question(s) we wanted to answer?

What did we find?

What are the implications of our findings?

We identified a cohort of young people with type 1 diabetes and severe and early neurodegenerative complications.

Could these neurodegenerative complications be linked to Wolfram syndrome?

We identified two new variants of WS1. All patients presented with early-onset diabetes, with an average age of onset of three years. There was a high prevalence of severe and early neurological complications, including optic atrophy (92%), deafness (80%), and urinary disorders (72%).

Ecuadorian patients under the age of 16 with non-autoimmune diabetes and optic atrophy should be investigated for WS1 to ensure they receive appropriate treatment.

## Graphical Abstract

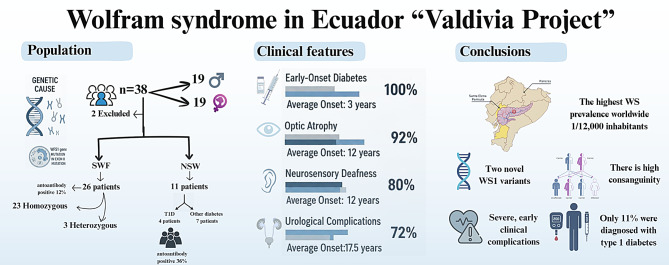

## Introduction

Wolfram syndrome (WS1) is a rare, autosomal recessive, neurodegenerative genetic disorder characterized by diabetes insipidus, early-onset non-autoimmune diabetes, optic atrophy, and sensorineural deafness, classically known by the acronym DIDMOAD [1]. This is often accompanied by urological disorders such as bladder dysfunction and loss of sphincter control, which is why some authors propose extending the term to DIDMOADUD [2]. WS1 is caused by mutation in gen *WFS1,* located on chromosome 4p16.1 [3].

The estimated prevalence of WS1 worldwide is 1 per 55,000 to 1 per 770,000 inhabitants, although it varies considerably depending on the population [4]. Rates of 1/1,000,000 have been reported in Spain [4]; 1/770,000 in the United Kingdom [5], 1/100,000 in North America; 1/68,000 in the Lebanese population and 1/54,478 in Sicily [2]. In Ecuador, at the time of this study, no epidemiological data or clinically confirmed cases of this disease had been reported.

Mutations in the *WFS1* gene affect the production and function of wolframin, an 890-amino acid transmembrane protein expressed mainly in the endoplasmic reticulum (ER) of cells in the central nervous system, pancreatic β cells, heart, and skeletal muscle [6–8]. Wolframin participates in maintaining calcium homeostasis, controlling ER stress, and regulating apoptosis [6, 9]. More than 200 mutations have been identified in the *WFS1* gene, most of them in exon 8, which encodes the C-terminal transmembrane domain, causing significant cellular dysfunction, especially in pancreatic β cells, leading to an early form of diabetes mellitus, and in neurons, causing progressive autosomal neurosensory degeneration [8].

This study aims to document, for the first time, clinically confirmed cases of Wolfram Syndrome in an admixture (European and indigenous Amerindian) population living along the coast of Santa Elena, Ecuador, and to report two new genetic variants, thereby contributing to a better epidemiological understanding of this disease in Latin America.

## Methodology

This manuscript was written following the Strengthening the Reporting of Observational studies in Epidemiology (STROBE) checklist for reporting observational studies [10].

This is a Cross-Sectional study conducted among individuals with early-onset insulin-dependent diabetes, residing in the Santa Elena Province, Ecuador. All participants with a prior diagnosis of type 1 diabetes (aged < 35) were included in the study.

Descriptive variables included age, gender, occupation, income, personal and family medical history, BMI, nutritional status, and blood pressure. Data collection was carried out by trained personnel from March 2023 to March 2025. At the start of the study, participants were evaluated for demographic, and anthropometric and nutritional characteristics. A detailed clinical history and family tree were obtained. Laboratory studies included: complete blood count, blood chemistry, lipid profile, liver function tests, electrolytes, HbA1c, C-peptide, antibodies (tyrosine phosphatase 2, islet cell or GAD-65), thyroid function tests, serum osmolarity, serum cortisol, IGF1, general urine analysis, albumin/creatinine ratio, FSH, LH, estradiol, testosterone, vitamin D3 and B12. In addition, genetic testing was performed: ORION (Whole Exome Sequencing – WES) using Massively Parallel Sequencing (Next Generation Sequencing) to determine whether participants had WS1 or not (Non-WS/NWS). Specialty evaluations included ophthalmological exam (visual fields, visual acuity, intraocular pressure, fundus exam, biomicroscopy, Ishihara test, macular tomography, optic nerve tomography (OCT), and orbital MRI); audiological study (otoscopy, bioimpedance testing, tympanometry, audiometry); cardiology evaluation and ECG; psychology, hand X-ray, and nephrology reno-vesical ultrasound; urological evaluation included the presence of urinary retention, incontinence, enuresis, and recurrent urinary tract infections.

For the diagnosis of diabetes insipidus, we used the following criteria: polyuria greater than 3 L/day, serum sodium > 145 mEq/L, urine specific gravity < 1.005, and plasma osmolality > 300 mOsm/kg, with quarterly evaluations.

Hypogonadism was defined according to the following criteria: hypergonadotropic (primary) hypogonadism was defined as total testosterone < 300 ng/dL with LH and/or FSH levels elevated above the upper limit for age. Hypogonadotropic (secondary) hypogonadism was defined as total testosterone < 300 ng/dL with low or inappropriately normal LH and/or FSH levels for age.

Hypothyroidism was assessed based on clinical history to identify patients with a prior diagnosis of hypothyroidism, along with evaluation of TSH and free T4 levels. Thyroid function tests were assessed annually and for dose adjustment.

Follow-up visits for each patient included an endocrinologist every 3 months, and with other specialists as needed.

No formal sample size calculation was conducted. The study used non-probabilistic convenience sampling, involving all type 1 diabetes patients treated at the health clinic of the non-profit organization “Fundación de Diabetes Juvenil del Ecuador” and “Futuro Valdivia”, who agreed to participate and signed assent/informed consent.

Descriptive statistics were used to compare genetic status (WS1 vs. NWS) by sex, and by age. Categorical variables were analyzed using the Pearson Chi-square test.

Approval was obtained from the Human Research Ethics Committee of Hospital Clínica Kennedy in Guayaquil, Ecuador, under code HCK-CEISH-2024–001 [11].

The data that support the findings are not publicly available due to participant privacy, but are available from the corresponding author upon reasonable request.

## Results

In the cohort of 38 participants (19 males) under 35 years old with a prior diagnosis of type 1 diabetes, genetic studies tested positive for WS1 in 26 patients. Among them, 8 (4 pairs) are siblings, and 5 are first cousins. Two patients were excluded: a 16-year-old female who met all clinical criteria for WS1—diabetes, blindness, deafness, chronic kidney disease due to bilateral hydronephrosis, cerebellar ataxia, and psychiatric disorders—died prematurely and did not undergo genetic testing; a second patient was excluded due to heterozygosity. This case may represent a WS-like phenotype and is therefore being analyzed separately.

A comparison between WS1 and NSW population characteristics is presented in Table 1. The duration of diabetes was twice as long in WS1; WS1 patients had lower BMI and lipid levels. WS1 group also showed higher albumin/creatinine ratio (150.2 mg/g vs. 24.03 mg/g) and elevated high-sensitivity CRP (1.05 mg/L vs. 0.4 mg/L). Diabetic nephropathy with GFR < 60 was found only in WS1 (12%). Albuminuria was 24% in WS1 vs. 9% in NWS. WS1 was genetically confirmed in 69% of the cohort. Among the remaining No-WS (NWS) group, only 4 patients had typical type 1diabetes (positive antibodies and low C-peptide). The average age was similar (17.5 years vs. 18 years), but WS1 patients were diagnosed with diabetes significantly earlier (3 ± 1.8 years vs. 11.5 ± 2.1 years). WS1 patients had significantly lower C-peptide levels and fewer positive antibodies (12% vs. 36%).

**Table 1:** Population characteristics

	WS1 n (%)	NWS n (%)
Number of participants	25	11
Male	15(60.0)	3(27.3)
Female	10(40.0)	8(72.7)
Average Age (years)	17.5	18.0
≥2 Positive Antibodies: (Tyrosine-phosphatase 2; islet cell antibodies; GDA-65)	3(12)	4(36)
Diabetes	25(100)	11(100)
Diabetic Retinopathy	2(8)	1(9)
ACR > 30 (mg/g)	6(24)	1(9)
CKD < 60 (ml/min/1.73 m^2^)	3(12)	0(0)
	**WS1**	**NWS**
Years with diabetes	14.5	5.8
BMI (kg/m^2^)	20.1	22.2
Early-onset Diabetes (years)	3.0	11.5
C-peptide (ng/ml)	0.14	1.05
HbA1c (A1C DCCT) (%), (mmol/mol)	9.3 78	8.7 72
GFT (ml/min/1.73 m^2^)	123.32	138.8
Triglycerides (mg/dl)	104.9	157.8
LDL (mg/dl)	110.7	133.2
HDL (mg/dl)	51.8	53.2
ACR (mg/g)	150.2	24.03
US-PCR (mg/L)	1.05	0.4
Urinary osmolarity (mOsml/L)	288.6	287

GDA = glutamic acid decarboxylase; ACR = albuminuria/creatiniuria ratio; CKD = chronic kidney disease; BMI = body mass index; GFT = glomerular filtration rate; US-PCR = ultra-sensitive reactive protein C

Table 2 shows genetic findings and characteristics of genetic variants. Twenty-five patients presented a new mutation in exon 8 classified as pathogenic. Of these, 23 were homozygous and 2 compound heterozygotes (both male). This new variant causes a frameshift mutation at codon 379 (aspartic acid to glycine), resulting in a premature STOP codon at position 164 (p.Asp379GlyfsTer164). Loss-of-function mutations in this gene are known to be pathogenic [12]. One compound heterozygote presented an additional new variant in exon 8, previously unreported. A third heterozygous patient had a variant of uncertain significance and was reported only as a carrier. There was no evidence of other type of monogenic diabetes among NWS patients.

**Table 2:** Genetic description of patients with Wolfram syndrome type 1

Gene	Exon/Intron Number	Variant Nomenclature	Genomic Nomenclature	Zygosity	Number	Classification	Inheritance	New Variant
*WFS1*	Exon 8	c.1135dupp.Asp379GlyfsTer164 [Depth- 62X/62X]	chr4:g.6302657dup	Homozygous	23	Probably pathogenic	Autosomal recessive	Yes
*WFS1*	Exon 8	c.2192T > Ap.Met731Lys [Depth-19X/57X]	chr4:g.6303714T > A	Heterozygous	1*	Uncertain meaning	Recessive	Yes
*WFS1*	Exon 4	c.397 G > Ap.Ala133Thr [Depth-61X/133X]	chr4:g.6290795 G > A	Heterozygous	1*	Probably pathogenic	Recessive	No
*WFS1*	Exon 8	c.1181A > Tp.Glu394Val [Depth- 64X/117X]	chr4:g.6302703A > T	Heterozygous	1	Uncertain significance	Recessive	No

*Compound heterozygotes

Table 3 shows WS1 clinical manifestations by gender over time. All patients presented with early-onset diabetes, with a mean age at diagnosis of 3 years. The mean HbA1c was 9.3% (78 mmol/mol), with only 12% of patients achieving levels below 7.5% (58 mmol/mol) and 48% exhibiting poor glycemic control with HbA1c values above 9% (75 mmol/mol). Optic atrophy developed in 92% of patients, with a mean age of onset of 12 years. Sensorineural hearing loss was observed in 80% of patients, predominantly among males, with a mean age of onset of 12 years. Urological tract involvement was identified in 72% of patients, also mainly affecting males, with a mean age of onset of 17.5 years, with the most frequently reported symptoms being bladder tenesmus and recurrent urinary tract infections. Post-void residual volume > 30% was documented in 32% of the patients; pelvic dilation and hydronephrosis were documented in 20 and 12% of the patients, respectively. Approximately 16% of the patients required intermittent bladder catheterization. Additionally, phimosis was found in 16% of the patients. No patient showed signs of diabetes insipidus. Serum osmolality was normal at the start (288.6 mOsm/L) and at the end (291.7 mOsm/L) of the study period. Hypothyroidism was found in one out of five patients, more commonly among females, and only in homozygous patients. Four out of five patients had been diagnosed with primary hypothyroidism and were under treatment with levothyroxine.

**Table 3:** WS1 clinical manifestations by gender over time

Variable	All n (%)	Male n (%)	Female n (%)	Average Age of Diagnosis (years)
Diabetes	25(100.0)	15(60.0)	10(40.0)	3(1–7)
Optic Atrophy	22(91.7)	15(68.2)	7(31.8)	12(8–17)
Neurosensorial Deafness	20(80.0)	13(65.0)	7(35.0)	12(9–22)
Urological Complications	18(72.2)	13(72.2)	5(27.8)	17.5(9–32)
Neurological Alterations	9(36.0)	4(44.4)	5(55.5)	Not reported
Hypogonadism	6(24.0)	6(100.0)	0	Not reported
Cataracts	5(20.0)	3(60.0)	2(40.0)	Not reported
Hypothyroidism	5(20.0)	2(40.0)	3 (60.0)	Not reported
Diabetes Insipidus	0	0	0	

Table 4 presents gonadal dysfunction for six patients. Hypergonadotropic hypogonadism (24%) was seen in heterozygous males (24%) with LH: 16. 2 mUI/mL (0.56 - 7.80), FSH 53.7 mUI/ml (1.27 - 19.30) and total testosterone 2.4 ng/ml (1.50 - 12.40).

**Table 4:** Gonadal dysfunction

	AGE (years)	LH mUI/mL 0.56 - 7.80	FSH mUI/mL 1.27 - 19.30	TESTOSTERONE ng/mL 1.50 - 12.40	TANNER
Patient 1	31	38.30	103.40	1.09	P2, G2
Patient 2	36	19.00	49.41	0.20	P1, G1
Patient 3	19	12.10	43.43	5.11	P3, G3
Patient 4	20	18.40	53.61	3.71	P3, G3
Patient 5	19	18.40	52.00	1.86	P2, G2
Patient 6	18	8.11	20.36	2.65	P4, G4

Neurological symptoms (cognitive decline, sensory neuropathy, sleep issues, etc.) were present in 36% or the patients. Two compound heterozygotes reported depression and suicide attempts. Two homozygous participants reported painful joint deformities.

**Fig. 1:**
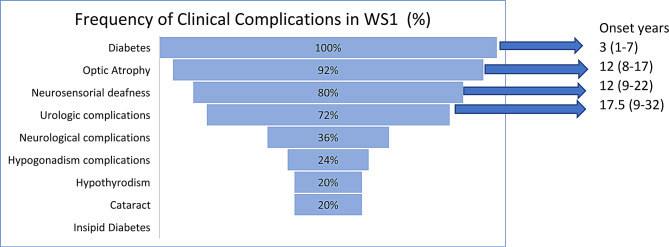
Percentage of clinical complications found in patients with Wolfram syndrome. It also indicates the average reported age of onset and the age range

Figure 1 presents the percentage of clinical complications found in patients with Wolfram syndrome and the average reported age of onset of clinical complications.

## Discussion

This study found a prevalence of 1 in 12,000 inhabitants in a relatively small coastal area in the province of Santa Elena, Ecuador—higher than reported rates in Sicily (1/54,478). The Ecuadorian population shows substantial degree of consanguinity: four sibling pairs and five first cousins, alike to the population profile describes in Sicily [2] a finding similar to that observed in Ecuador.

This study reports two previously undescribed variants, both located in exon 8: the first, c.1135dupp.Asp379GlyfsTer164 [Depth-83X/83X], was found in 100% of patients with WS1, causing a mutation in the frameshift of codon 379 of aspartic acid by glycine residue, which causes a premature stop in the sequential flow at codon 164 p.Asp379GlyfsTer164. Loss-of-function variants in this gene are recognized as pathogenic [12]. The second new variant c.2192T > Ap.Met731Lys[Depth-19X/57X], previously unreported in the literature, is of undetermined clinical significance and was found in a compound heterozygous male patient carrying both new variants [13].

In addition, two known variants are described, one in exon 4 class 4 (probably pathogenic) in a compound heterozygote and another in exon 8 class 3 (clinical significance undetermined) [13]. These two known variants are among the more than 200 mutations published in the scientific literature in patients with WS1, most of which are located in exon 8 [8, 13].

In the Ecuadorian cohort, no cases of diabetes insipidus (DI) were found – a feature of these new variants – all patients carry the same mutation (described as novel), which may explain the absence of diabetes insipidus; however, active surveillance for diabetes insipidus is being maintained. In contrast, the UK cohort reported DI in 73% of 45 WS1 patients [5]. This cohort exhibited very early onset of diabetes (age 3), followed by optic atrophy in 92%, sensorineural hearing loss in 80%, urinary tract issues in 72%, neurological disorders in 36%, hypogonadism in 24%, hypothyroidism in 20%, and cataracts in 20%. The UK cohort reported non-autoimmune diabetes at an average age of 6, followed by optic atrophy (100%), sensorineural hearing loss (62%), urinary tract issues (58%), neurological disorders (62%), and diabetes insipidus (73%) [5].

A strength of this study is the antibody testing (tyrosine phosphatase 2, islet cell, and GAD-65). In the Ecuadorian WS1 group, 12% tested positive for antibodies, consistent with a German multicenter study reporting 10% [14]. In the NWS group, 36% were antibody-positive, while the same study reported 86% [14]. This is crucial for identifying other diabetes types that may require different treatment. All patients in the cohort are being treated with a basal–bolus insulin regimen. The incorporation of continuous glucose monitoring (CGM) in the second phase of the study will allow treatment adjustments, particularly in patients with negative autoantibodies and normal C-peptide levels, who represent 63% of the NWS group.

Optic atrophy, the second major clinical feature of WS1, was observed in 92% of patients in this cohort, with a mean age of onset of 12 years. It is important to note that two participants have not yet received a conclusive diagnosis due to their young age. Most patients initially reported impairment in color vision at approximately 11 years of age [1], which is consistent with findings from the UK cohort, where the mean age of onset was also 11 years [5].

Sensorineural hearing loss was present in 80% of participants, with a mean age of onset at 11.5 years. Literature reports rates between 62 and 65%, with onset ranging from 12.5 [2, 5] to 16 years [2, 8]. Hearing loss can be congenital or develop during adolescence [1].

Urinary tract complications—including glomerular hyperfiltration, hydronephrosis, tenesmus, and neurogenic bladder—were found in 73% of patients, appearing at a mean age of 17 years. Similar rates (60–90%) have been reported in the literature [1], with onset around age 20 [9]. This high prevalence has prompted some to propose redefining the condition as DIDMOADUD [2, 15]. Neurological disorders—such as epilepsy, ataxia, severe depression, psychosis, impulsivity, smell disturbances, sleep issues, aggression, and central apnea—tend to appear later in WS patients [9]. In this cohort, 36% of the patients had symptoms such as epilepsy, cognitive decline, nystagmus, ptosis, corticospinal syndrome, daytime hypersomnia, headache, and sensory neuropathy. These may increase over time. Literature reports similar rates between 32% [15] to 72% [1].

Severe psychiatric illnesses are significantly more common in heterozygous carriers, who have a 26-fold higher risk of hospitalization and increased suicide risk compared to spousal controls [16]. Psychiatric issues typically emerge between ages 17 and 23 [9]. Two heterozygous patients in our cohort developed depression at 25 years of age, and another patient had a history of two suicide attempts at 20 years of age.

Other findings include primary hypogonadism in 24% of male participants consistent with results reported in the literature [2, 17] and hypothyroidism, cataracts, and delayed menarche [2] in one out of five female patients.

Compound heterozygous patients demonstrated better glycemic control (lower HbA1c), fewer clinical manifestations, and a higher prevalence of psychiatric disorders compared with homozygous patients, consistent with findings from previous studies [16].

Most studies report a lower incidence of microvascular complications in patients with WS1 compared with those with type 1 diabetes [18]. However, in this Ecuadorian cohort, WS1 patients exhibited higher rates of proteinuria (24% vs. 9%) and nephropathy (12% vs. 0%) compared with NWS patients. The prevalence of retinopathy was slightly lower in the WS1 group (8%) than in NWS patients (9%). Glycemic control was poorer among WS1 patients, with a mean HbA1c of 9.3% (78 mmol/mol), compared with 8.7% (72 mmol/mol) in the NWS group. These findings contrast with reports from a French cohort, in which WS1 patients demonstrated better glycemic control than individuals with type 1 diabetes (mean HbA1c 7.7% [61 mmol/mol] vs. 8.9% [74 mmol/mol]) and a lower prevalence of microvascular complications, including retinopathy (8% vs. 27%), proteinuria (8% vs. 19%), and nephropathy (8% vs. 27%) [18].

The earlier onset of diabetes in the Ecuadorian cohort leads to greater deterioration of pancreatic beta cells mediated by ER stress, which could be the determining factor in the higher and earlier prevalence of microvascular complications observed.

## Conclusions

The presence of two new variants of WS1 has been demonstrated, for the first time, in a young, admixture population living in a relatively small coastal area of Ecuador. It represents the highest prevalence of WS1 in the world and the earliest onset of non-autoimmune diabetes. The founder effect is a mechanism of genetic drift that reduces genetic variability in small populations and will be investigated in subsequent phases of the study.

Patients have a high prevalence of severe and early neurodegenerative features, as well as a higher percentage of microvascular complications. WS1 should be investigated in the presence of non-autoimmune diabetes and optic atrophy in patients under 16 years of age.

Given that only 11% of patients meet the diagnostic criteria for type 1 diabetes, it is imperative to phenotype diabetes in populations residing in the coastal area of Ecuador and provide appropriate treatment. Whole-exome sequencing did not reveal other forms of early-onset, non-autoimmune diabetes; however, the presence of a deep intronic variant cannot be excluded.

## Recommendations

Further research is needed to genetically characterize relatives of the studied cohort and to provide appropriate genetic counseling to affected families. The implementation of diabetes management technologies is recommended to improve glycemic control, reduce the risk of hypoglycemia, and limit the development of microvascular complications.

In addition, the development of standardized protocols for the diagnosis and management of WS1 is essential for populations residing in the regions studied. Although no disease-modifying therapies are currently available to slow disease progression, the primary objective remains the provision of comprehensive, patient-centered care. This requires the establishment of multidisciplinary teams involving healthcare professionals, members of the scientific community, social organizations, and governmental authorities, with the goal of improving the quality of life of individuals living with WS1 and their caregivers.

## Declarations

### Prior presentation

Preliminary results of this study were presented in an abstract format at the American Diabetes Association’s 85th Scientific Sessions, June 20-23, 2025 in Chicago, Illinois, USA (https://www.abstractsonline.com/dashboard/login.asp?aId=318%26targetMKey=491c7de7-cf6e-4a71-9343-a25943b25ad2%26targetMod=notifyMessages).
